# Lymphoplasmacytic lymphoma with IgG-λ and free λ light chain paraprotein: A case report

**DOI:** 10.1097/MD.0000000000044847

**Published:** 2025-10-10

**Authors:** Luting Wang, Qiuxia Wan, Lina Lu, Shuya Cao, Beibei Liu, Suyun Wang

**Affiliations:** a Department of Hematology, Shenzhen Longhua District Central Hospital, Shenzhen, Guangdong, China.

**Keywords:** free λ light chain, IgG-λ, lymphoplasmacytic lymphoma

## Abstract

**Rationale:**

Lymphoplasmacytic lymphoma (LPL) represents a rare, indolent form of B-cell neoplasm, with non–immunoglobulin M subtypes, including the immunoglobulin G (IgG)-λ variant, being notably uncommon. In this report, we document a case of LPL distinguished by the presence of monoclonal IgG-λ immunoglobulin and free λ light chains, alongside its distinctive molecular characteristics and therapeutic outcomes.

**Patient concerns:**

A 58-year-old male presented with fatigue, leukocytosis (75.45 × 10^9^/L, 88.8% lymphocytes), lymphadenopathy, and splenomegaly. Serum studies detected an IgG-λ monoclonal protein (6.74 g/L) with concurrent elevation of free λ light chains. Bone marrow biopsy revealed marked hypercellularity, with lymphocytes comprising 80% of nucleated cells (predominantly plasmacytoid lymphocytes) and 5% plasma cell clusters. Genetic testing identified mutations in MYD88, CXCR4, and IGHV, along with trisomy 12 and del(13q14).

**Diagnoses:**

He was diagnosed with LPL with IgG-λ monoclonal immunoglobulin and free λ light chains, classified as low risk per the Revised International Prognostic Scoring System.

**Interventions:**

The patient received 3 cycles of bendamustine plus rituximab therapy.

**Outcomes:**

The blood cell count returned to normal and the spleen and lymph nodes were significantly reduced. Serum M protein levels decreased, and no obvious increase in B lymphocytes or plasma cells was found in the bone marrow. The patient achieved partial remission.

**Lessons:**

This case highlights the diagnostic and therapeutic challenges associated with the IgG-λ subtype of LPL, an uncommon variant of this rare malignancy. This report provides valuable insights into the clinical presentation, pathological features, molecular alterations, and treatment outcomes of this rare disease.

## 1. Introduction

Lymphoplasmacytic lymphoma (LPL) is a rare indolent B-cell lymphoma (<2% of non-Hodgkin lymphomas), defined by bone marrow infiltration of lymphocytes, plasmacytoid lymphocytes, and plasma cells, often accompanied by monoclonal gammopathy.^[1]^ Most cases are associated with immunoglobulin M (IgM) (lymphoplasmacytic lymphoma/Waldenström macroglobulinemia, LPL/WM), while non-IgM subtypes (immunoglobulin G [IgG]/immunoglobulin A) account for only 5% to 10%.^[2]^ Among these, the occurrence of LPL with IgG-λ monoclonal immunoglobulin and concomitant free λ light chains is notably uncommon, and there is a paucity of data regarding its clinical, pathological, and molecular characteristics.^[3]^ In this report, we document a case of LPL exhibiting mutations in MYD88 and CXCR4 genes, emphasizing the diagnostic indicators and therapeutic responsiveness to facilitate a deeper comprehension of this unique subtype.

## 2. Case presentation

**Figure 1.**
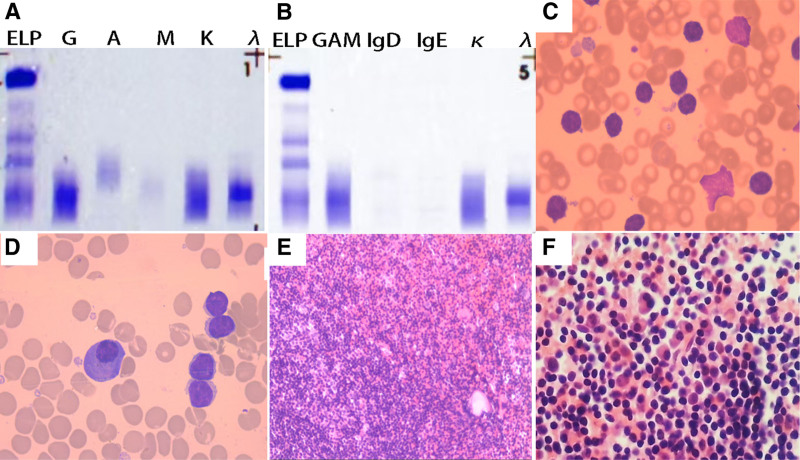
Serum immunofixation and bone marrow pathology. (A, B) Serum immunofixation electrophoresis demonstrates monoclonal IgG-λ paraprotein with free λ light chains (A: IgG/IgA/IgM; B: IgD/IgE). (C, D) Bone marrow aspirate (1000×) reveals hypercellularity with lymphocytes accounting for 85.5% of nucleated cells, including 30% atypical lymphocytes with plasmacytoid features; plasma cells are present. (E, F) Bone marrow biopsy (10×/40×) shows marked hypercellularity, with lymphocytes accounting for the majority (≈80% of nucleated cells), predominantly plasmacytoid lymphocytes, and plasma cell clusters (≈5%). IgA = immunoglobulin A, IgD = immunoglobulin D, IgE = immunoglobulin E, IgG = immunoglobulin G, IgM = immunoglobulin M.

A 58-year-old male patient was admitted with a 1-month history of fatigue and a sudden increase in white blood cell count (75.45 × 10⁹/L, with 88.8% lymphocytes). He negated the presence of B symptoms, which include fever, night sweats, and weight loss, and reported no significant medical or familial history. The physical examination disclosed palpable lymphadenopathy in the axillary and inguinal regions. Imaging studies corroborated the presence of splenomegaly, with measurements of 46 mm in thickness and 120 mm in length. Additionally, computed tomography of the chest and abdomen revealed multiple enlarged lymph nodes, with the largest having a short-axis diameter of 11 mm. Serological investigations revealed the presence of an M-protein band (6.74 g/L) corresponding to an IgG-λ monoclonal immunoglobulin, accompanied by elevated levels of free λ light chains (serum IgG: 24.85 g/L; λ light chain: 3.48 g/L; serum free λ: 153.49 mg/L, serum Fκ/Fλ ratio: 0.1978; urine free λ: 27.17 mg/L, urine Fκ/Fλ ratio: 3.1984), whereas urinary protein was undetected (Fig. 1A, B).

The evaluation of the bone marrow disclosed a significant increase in cellularity. The aspirate (1000×) exhibited that lymphocytes constituted 85.5% of the nucleated cells, with 30% of these being atypical in nature – principally plasmacytoid lymphocytes, which are distinguished by their rounded to oval nuclei, finely granular chromatin, inconspicuous nucleoli, and abundant basophilic cytoplasm with marginally irregular contours – alongside a dispersed population of plasma cells (Fig. 1C, D). The bone marrow biopsy (10×/40×) revealed a marked suppression of normal hematopoietic activity. Additionally, there was a substantial increase in the number of lymphocytes, constituting roughly 80% of the observed cells. The lymphocytes were predominantly plasmacytoid in nature and were found to be diffusely situated in proximity to non-trabecular bone regions. Plasma cells were present in small clusters and aggregates, comprising approximately 5% of the total cell population (Fig. 1E, F).

**Figure 2.**
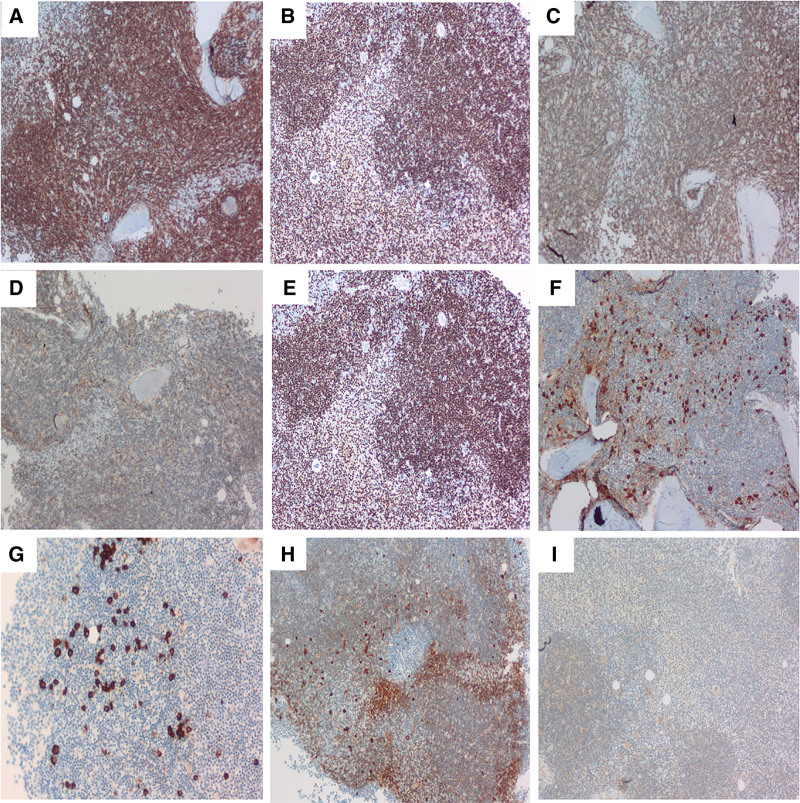
Bone marrow immunohistochemistry (10×). (A–E) B-cell lineage markers (CD19, CD20, CD22, PAX5, CD200) show diffuse positivity (CD19/CD20/CD22/PAX5) and partial positivity (CD200), confirming B-cell derivation. (F, G) Plasma cell markers (CD38, CD138) exhibit scattered and clustered positivity, indicating plasma cell differentiation. (H) Immunoglobulin light chain analysis demonstrates restricted λ expression. (I) MYD88 shows partial weak positivity, supporting LPL diagnosis. LPL = lymphoplasmacytic lymphoma.

**Figure 3.**
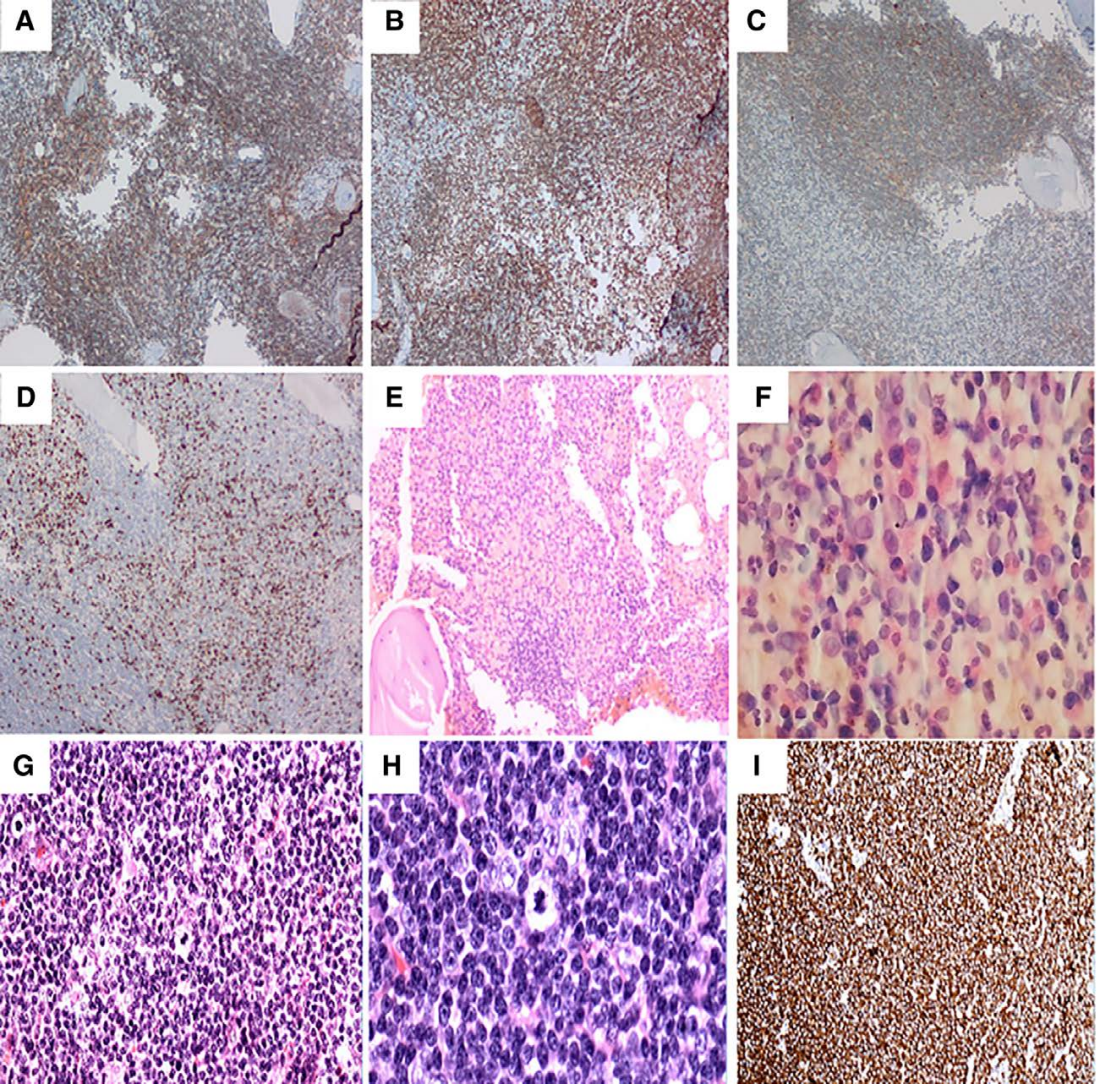
Bone marrow and lymph node analysis. (A–D) Bone marrow biopsy immunohistochemistry (10×) displays (A) diffuse IgD positivity, (B) patchy BCL-2 positivity, (C) heterogeneous CD25 positivity, and (D) a Ki-67 proliferation index of 5%. (E, F) Post-bendamustine-rituximab (BR) therapy, bone marrow tissue (10×/40×) reveals no residual lymphoma cells. (G, H) Lymph node sections (20×/40×) demonstrate diffuse architectural effacement by atypical lymphoid cells. (I) CD20 immunohistochemistry (20×) confirms B-cell lineage, supporting B-cell lymphoma diagnosis. BR = bendamustine plus rituximab, IgD = immunoglobulin D.

Immunohistochemical analysis of the bone marrow biopsy revealed widespread expression of B-cell lineage markers, including CD19, CD20, CD22, and PAX5, thereby affirming the clonal population’s derivation from B-cells. Partial positivity for CD200 and a weak partial positivity for MYD88 were also observed. It is crucial to note that a restriction of the λ light chain was observed, which is consistent with the clonal nature of the lymphoid infiltrate. This is corroborated by the scattered or clustered positivity for plasma cell markers (CD38, CD138), which further confirms plasmacytic differentiation within the infiltrate (Fig. 2). The proliferation index, as indicated by Ki-67 (Fig. 3A–D), was determined to be 5%, and the expression of P53 was found to be weakly present in 1% of the cells. To rule out alternative lymphoid neoplasms, additional markers were found to be negative: the T-cell marker CD3 (excluding T-cell lineage), and markers specific to B-cell lymphoma subtypes (CD5, CD10, CD23, Cyclin-D1, SOX-11), which facilitated differentiation from other B-cell malignancies.

Flow cytometric analysis of the bone marrow aspirate confirmed the presence of a mature B-cell lymphoma phenotype characterized by the absence of CD5 and CD10 markers, consistent with the immunophenotypic profile of LPL. The lymph node biopsy paralleled the bone marrow results, disclosing an indolent small B-cell lymphoma characterized by widespread positivity for B-cell markers (CD20, CD79a, BCL-2, PAX5), partial positivity for MUM1 (suggestive of plasmacytic differentiation), and localized CD138 expression within clusters (indicative of plasma cell components). Ki-67 was focally expressed in 15% of cells in hotspots, with P53 expression in 5% of cells; negativity for BCL6, CD10, CD43, CD5, and Cyclin-D1 further supported the exclusion of other B-cell lymphoma subtypes (Fig. 3G–I).

**Figure 4.**
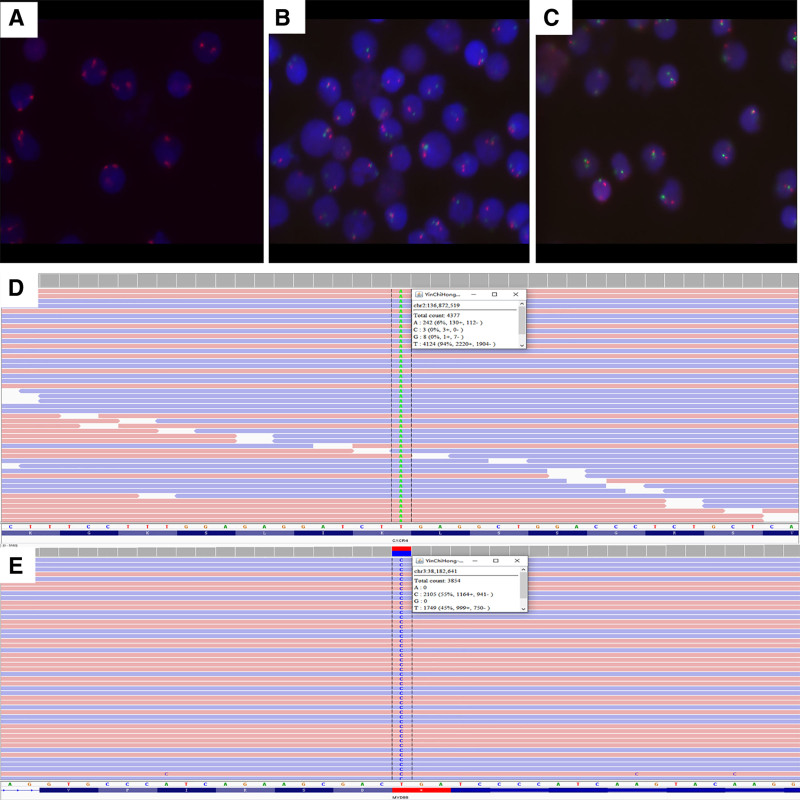
Molecular cytogenetics. (A–C) Fluorescence in situ hybridization (FISH) analysis detects trisomy 12 and del(13q14; RB1/D13S319).(D, E) Next-generation sequencing (NGS) identifies CXCR4 and MYD88 mutations. FISH = fluorescence in situ hybridization, NGS = next-generation sequencing.

Molecular and cytogenetic studies by next-generation sequencing identified mutations in MYD88, CXCR4, and IGHV, while fluorescence in situ hybridization detected trisomy 12 and deletions at 13q14 (involving RB1 and D13S319 loci; Fig. 4).

The patient was diagnosed with LPL exhibiting monoclonal immunoglobulin of the IgG-λ subtype, accompanied by free λ light chains. Categorized as low-risk according to the revised International Prognostic Scoring System with a score of 1, the patient underwent 3 cycles of bendamustine plus rituximab (BR) therapy, resulting in partial remission. The white blood cell count was normalized to 4.22 × 10⁹/L, lymphadenopathy and splenomegaly regressed, serum M-protein levels decreased (from 6.74 to 6.61 g/L), and a follow-up bone marrow biopsy indicated no significant malignant infiltration (Fig. 3E, F).

## 3. Discussion

The case of LPL characterized by the presence of IgG-λ monoclonal immunoglobulin and free λ light chains underscores the diagnostic complexities associated with non-IgM subtypes. In comparison to LPL/WM, which is often accompanied by hyperviscosity syndrome, the non-IgM variants frequently exhibit more subtle symptoms (such as fatigue and leukocytosis), potentially leading to a delay in diagnosis.^[4]^ Confirmation relied on the integration of clinical, morphological (mixed plasmacytoid lymphocytes/plasma cells in bone marrow), immunophenotypic (B-cell markers with λ light chain restriction), and molecular data to exclude other B-cell neoplasms.

The molecular profile, characterized by concurrent mutations in MYD88 and CXCR4, provides significant insights. The MYD88 L265P mutation, which acts as a driver in over 90% of IgM-LPL cases through NF-κB activation,^[5,6]^ exhibits a lower prevalence in non-IgM LPL, affecting 40% to 70% of cases,^[7]^ a finding that is consistent with our observations. CXCR4 mutations, present in 30% of LPL,^[8]^ are known to reduce sensitivity to Bruton tyrosine kinase (BTK) inhibitors (e.g., ibrutinib) and alkylating agents like bendamustine.^[9]^ This likely explains the modest serum M-protein reduction (6.74–6.61 g/L) and partial remission with BR therapy in this case.

Additional genetic characteristics render the situation more complex: mutations in the IGHV gene are detected in over 97% of cases associated with LPL/WM,^[10,11]^ substantiating the clonal B-cell origin, with instances of MYD88 L265P typically exhibiting elevated rates of IGHV hypermutation, which impacts the disease’s progression. Cytogenetic abnormalities, such as the deletion of 13q14 and trisomy 12 – prevalent in 5.3% and 7.6% of Chinese LPL cohorts respectively^[12,13]^ – may interact with these mutations to alter the progression of the disease, although their influence on non-IgM subtypes necessitates additional investigation.

In the therapeutic context, non-IgM LPL lacks established standardized guidelines, and thus strategies are often derived from those developed for LPL/WM. The BR regimen, alongside BTK inhibitors, are considered first-line treatment options. Our patient exhibited a partial response to the BR regimen. However, the presence of CXCR4 mutations can hinder the efficacy of BTK inhibitors, as supported by studies.^[14,15]^ Consequently, BCL-2 inhibitors, such as venetoclax, which are unaffected by the CXCR4 mutation status,^[16]^ present as a viable additional treatment option for this particular case.

In summation, this case underscores the imperative of utilizing a multimodal diagnostic methodology and conducting molecular profiling in non-IgM LPL to facilitate the development of personalized therapeutic strategies. This is particularly relevant in light of the substantial impact that mutations, such as those affecting CXCR4, can exert on therapeutic outcomes. It is imperative that further extensive research be conducted to refine the management protocols for these rare subtypes.

## 4. Conclusions

Knowledge regarding how the interplay between molecular mutations (MYD88, CXCR4, IGHV) and cytogenetic abnormalities impacts therapy response and the development of personalized targeted therapy in non-IgM LPL is still scarce. The importance of this case cannot be overemphasized for future studies on rare variants of lymphoma.

## Author contributions

**Conceptualization:** Luting Wang, Qiuxia Wan.

**Data curation:** Luting Wang, Shuya Cao.

**Formal analysis:** Luting Wang, Lina Lu, Beibei Liu.

**Methodology:** Suyun Wang.

**Project administration:** Luting Wang.

**Supervision:** Suyun Wang.

**Writing – original draft:** Luting Wang.

**Writing – review & editing:** Suyun Wang.
